# The prohibitin protein complex promotes mitochondrial stabilization and cell survival in hematologic malignancies

**DOI:** 10.18632/oncotarget.18920

**Published:** 2017-07-01

**Authors:** Jeremy A. Ross, Elisa Robles-Escajeda, Derrick M. Oaxaca, Diana L. Padilla, Robert A. Kirken

**Affiliations:** ^1^ Department of Biological Sciences and Border Biomedical Research Center, The University of Texas at El Paso, El Paso, TX 79968, USA

**Keywords:** prohibitin, lymphoma, leukemia, mitochondria, apoptosis

## Abstract

Prohibitins (PHB1 and PHB2) have been proposed to play important roles in cancer development and progression, however their oncogenic mechanism of action has not been fully elucidated. Previously, we showed that the PHB1 and PHB2 protein complex is required for mitochondrial homeostasis and survival of normal human lymphocytes. In this study, novel evidence is provided that indicates mitochondrial prohibitins are overexpressed in hematologic tumor cells and promote cell survival under conditions of oxidative stress. Immunofluorescent confocal microscopy revealed both proteins to be primarily confined to mitochondria in primary patient lymphoid and myeloid tumor cells and tumor cell lines, including Kit225 cells. Subsequently, siRNA-mediated knockdown of PHB1 and PHB2 in Kit225 cells significantly enhanced sensitivity to H_2_O_2_-induced cell death, suggesting a protective or anti-apoptotic function in hematologic malignancies. Indeed, PHB1 and PHB2 protein levels were significantly higher in tumor cells isolated from leukemia and lymphoma patients compared to PBMCs from healthy donors. These findings suggest that PHB1 and PHB2 are upregulated during tumorigenesis to maintain mitochondrial integrity and therefore may serve as novel biomarkers and molecular targets for therapeutic intervention in certain types of hematologic malignancies.

## INTRODUCTION

The prohibitin (PHB) family is composed of two members, PHB1 and PHB2. PHB1, formerly known as BAP32 and its homolog PHB2, previously known as BAP37 or REA, are pleiotropic proteins with multiple functions [1]. Genetic deletion of PHB1 and PHB2 is embryonically lethal in mice indicating that these proteins perform an essential role in embryonic development [2, 3] and are known to be evolutionary conserved with homologues found in organisms from yeast to man [4].

Prohibitins have been reported to elicit multiple functions that may be defined by their cellular localization and cell type. The multiple functions attributed to prohibitins include nuclear transcription, plasma membrane lipid scaffold protein, and in the mitochondria as a regulator of mitochondrial morphogenesis and apoptosis [5–14]. Despite these diverse biological roles, the function of prohibitin proteins in cancer remains poorly understood [15].

PHB1 was originally described as a tumor suppressor for its ability to inhibit cell proliferation [16]. This effect was later attributed to the 3′-UTR and not the PHB1 protein [17], however, reports indicate PHB1 can inhibit proliferation through interaction with the cell cycle checkpoint molecules E2F [18], p53 [19], and pRb [20]. PHB2, also known as repressor of estrogen receptor activity, was shown to directly interact with and inhibit the transcriptional activity of the estrogen receptor [2]. Moreover, previous studies have shown that phytochemical flavaglines and the synthetic fluorinated small molecule fluorizoline target prohibitins causing disruption of the Raf-MEK-ERK signaling pathway and induction of apoptosis, respectively [21–23]. Taken together, the array of prohibitin activity suggests these proteins might be attractive therapeutic targets for a variety of disease states, including inflammation, obesity and cancer, however a better understanding of their cell dependent function appears to be essential [24].

Prohibitins have been implicated in cancer progression through regulation of key cell signaling pathways known to induce cell proliferation, resistance and metastasis, including the Ras/Raf/MEK/ERK, PI3K/AKT and TGF-β [1, 25–27]. As a consequence, high levels of prohibitin expression have been demonstrated in several transformed cells and in many primary human cancers, including endometrial adenocarcinoma [28], hepatocellular carcinoma [29], gastric cancer [30], esophageal cancer [31], bladder cancer [32], and breast cancer [33], however their role in hematologic malignancies and tumorigenic mechanisms of action have not been fully elucidated. Evidence suggests that cellular localization is a key determinant of prohibitin function [1, 31, 34, 35]. Previously, we showed that the PHB1 and PHB2 protein complex is required for mitochondrial homeostasis and survival of normal human lymphocytes [36].

Yeast molecular genetics has played a key role in understanding prohibitin function. The PHB1 and PHB2 homologues in *Saccharomyces cerevisiae* form a high molecular weight complex within the inner mitochondrial membrane and are proposed to function as chaperones for newly imported proteins including electron transport enzymes [7, 8, 37]. Moreover, enhanced oxidative stress has been associated with PHB expression. In endothelial cells, down-regulation of PHB resulted in increased mitochondrial reactive oxygen species (ROS) production and cellular senescence [16], whilst over-expression of PHB in intestinal epithelial cells ameliorated oxidative stress in inflammatory bowel disease [17]. Under physiological conditions, levels of intracellular reactive oxygen species (ROS) are maintained as byproducts of normal metabolism in eukaryotic cells. These normally low ROS concentrations have important roles in cell signaling and homeostasis [38]. However, oxidative stress can occur when the equilibrium between the generation of ROS and their detoxification by antioxidant proteins is disrupted. Oxidative stress disturbs crucial cellular functions and has been related in a wide spectrum of diseases, including chronic inflammation and oncogenesis [39, 40]. Indeed, increased levels of ROS are persistently elevated in several types of cancers [39].

The present study was initiated to determine the role of PHB1 and PHB2 in T- and B-cell malignancies. We provide novel evidence that PHB1 and PHB2 are upregulated in hematologic tumor cells to maintain mitochondrial integrity and protect against oxidative stress-induced cell death. These findings provide further evidence regarding the importance of PHB1 and PHB2 in lymphocyte function and dysfunction.

## RESULTS

### PHB1 and PHB2 are overexpressed in human lymphoid and myeloid tumor cell lines

**Figure 1:**
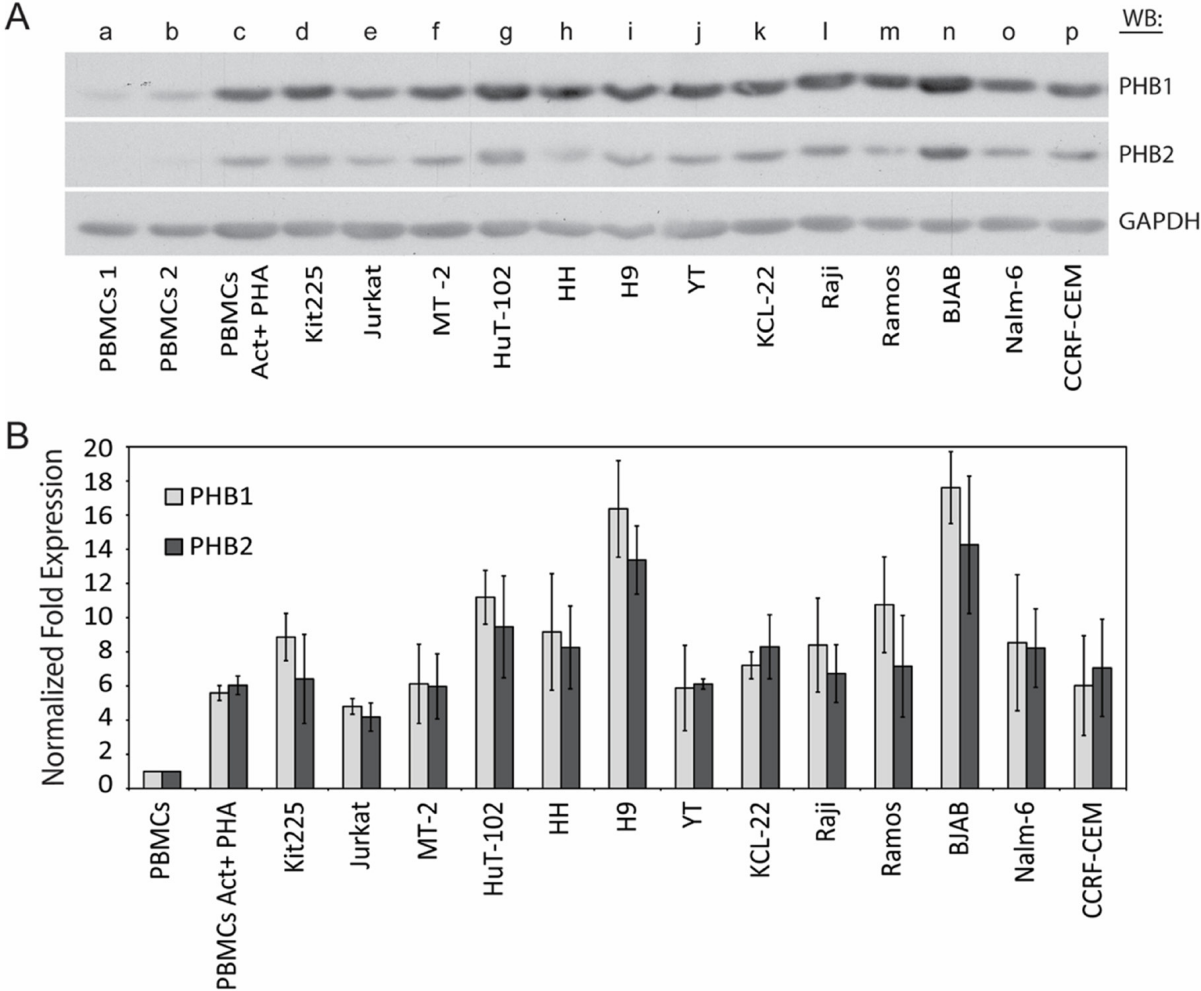
PHB1 and PHB2 protein expression in human lymphoid and myeloid derived tumor cell lines **(A)** Naïve (lane a and b) or PHA activated primary human PBMCs (lane c), CLL T cell line Kit225 (lane d), ALL T cell line Jurkat (lane e), HTLV-1 transformed T cell lines MT-2 and Hut102 (lane f and g), CTCL cell lines HH and H9 (lane h and i), NK-like lymphoma cell line YT (lane j), CML cell line KCL-22 (lane k), Burkitt’s lymphoma cell lines Raji, Ramos and BJAB (lane l, m and n), pre-B-ALL cell line NALM-6 (lane o), and ALL cell line CCRF-CEM (lane p) cell lysates (10 µg) were separated by 10% SDS-PAGE and subjected to Western blot analysis with antibodies directed to PHB1, PHB2 or GAPDH. (**B**) PHB1 and PHB2 band intensities were normalized to GAPDH using densitometric analysis and the fold increase plotted for each cell type. Values represent the mean ± S.D. of three independent experiments.

PHB1 and PHB2 protein levels have been reported to be higher in several transformed cells as compared to their non-transformed counterparts. To test this notion within hematologic malignancies, the expression levels of PHB1 and PHB2 were investigated in a panel of lymphoid and myeloid-derived tumor cell lines. As shown (Figure 1A and 1B), normal naïve (lane a and b) and PHA-activated (lane c) human PBMCs were compared to the chronic lymphocytic leukemia T-cell line Kit225 (lane d), acute lymphoblastic leukemia T-cell line Jurkat (lane e), HTLV-1 transformed T-cell lines MT-2 and Hut102 (lane f and g), cutaneous T-cell lymphoma cell lines HH and H9 (lane h and i), NK-like acute lymphoblastic lymphoma and thymoma cell line YT (lane j), chronic myelogenous leukemia cell line KCL-22 (lane k), Burkitt’s lymphoma cell lines Raji, Ramos and BJAB (lane l, m and n), pre-B acute lymphoblastic leukemia cell line NALM-6 (lane o), and acute lymphocytic leukemia cell line CCRF-CEM (lane p) by Western blot analysis of total cell lysate (Figure 1A). The membrane was stripped and reprobed for GAPDH to confirm equal loading. Consistent with our previous findings, densitometric analysis indicated PHB1 and PHB2 protein levels were upregulated upon activation of primary human PBMCs (5.34 and 5.44 average fold increase for PHB1 and PHB2 respectively) (Figure 1B) [36]. Compared to naive primary human PBMCs, PHB1 and PHB2 protein levels were 4.3 to 18.4 and 3.6 to 18.4 fold higher (*p <* 0.05) in the tumor cell lines, respectively. Taken together, PHB1 and PHB2 proteins are overexpressed in lymphoid and myeloid tumor cell lines compared to normal naïve and activated primary human PBMCs.

### PHB1 and PHB2 co-localize to the mitochondria of Kit225 cells

Identifying the subcellular localization of PHB1 and PHB2 in hematologic tumor cells is important to understanding their function in hematologic cell transformation, as well as the general mechanism of action in normal human lymphocytes. To determine their localization, dual labeled immunofluorescent confocal microscopy was utilized on the model cell line Kit225. The interleukin-2 dependent Kit225 cell line was established from a patient diagnosed with T-cell chronic lymphocytic leukemia [41].

**Figure 2:**
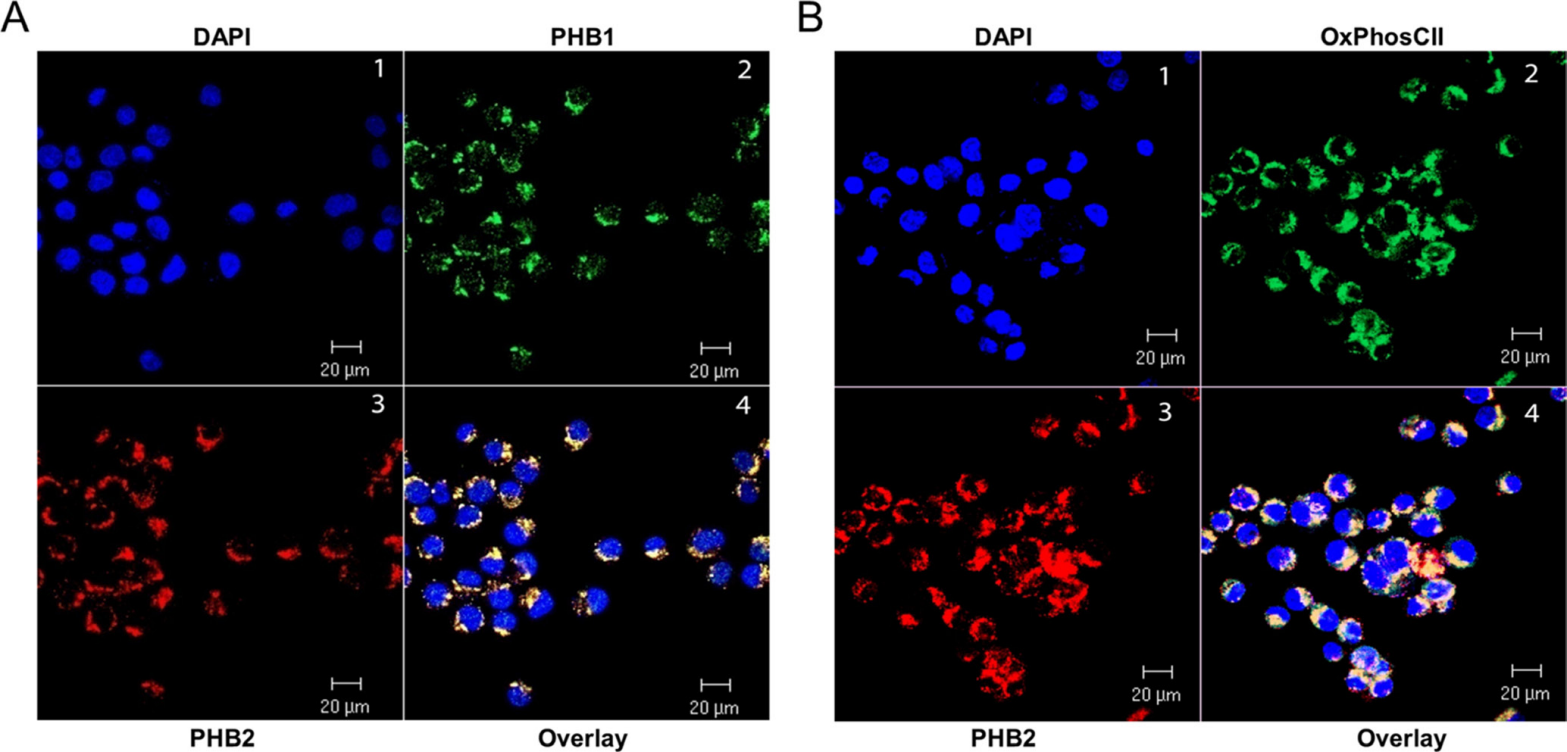
PHB1 and PHB2 co-localize to mitochondria of Kit225 cells Kit225 cells were cytocentrifuged onto glass slides and subjected to analysis by immunofluorescent confocal microscopy. (**A**) PHB subcellular localization was determined by staining the nucleus with DAPI (panel 1); PHB1-Cy2 (panel 2) and PHB2-Cy3 (panel 3) and overlay (panel 4). (**B**) Nuclear staining with DAPI (panel 1), OxPhos CII-Cy2 (panel 2), PHB2-Cy3 (panel 3) and overlay (panel 4) were detected in Kit225 cells using immunofluorescent confocal microscopy.

The nucleus was identified with the DNA binding fluorescent stain DAPI (Figure 2A, Panel 1). PHB1 (Panel 2) and PHB2 (Panel 3) co-localize primarily to peri-nuclear regions (Panel 4) with a specific punctuate staining pattern in Kit225 cells. There were no detectable levels of PHB1 or PHB2 at the plasma membrane or in the nucleus. To determine whether the prohibitin complex can be localized to mitochondria in Kit225 cells, the inner mitochondrial membrane marker protein OxPhos CII was utilized (Figure 2B). Dual immunofluorescent confocal microscopy detecting PHB2 (Panel 3) and OxPhos CII (Panel 2) in tandem with DAPI (Panel 1) showed co-localization of PHB2 with OxPhos CII (Panel 4). Taken together, PHB1 and PHB2 were identified to be primarily confined to the mitochondria in lymphoid tumor cell lines, including Kit225 cells.

### PHB1 and PHB2 are upregulated during ROS-mediated apoptosis of Kit225 cells, while reduced PHB1/2 expression results in increase cell death during oxidative stress

Accumulating evidence suggests that prohibitins play a role in preventing oxidative stress in an array of cell types [42, 43]. Previously, we showed PHB1 and PHB2 protein levels were upregulated during cytokine deprivation-mediated apoptosis in Kit225 cells, suggesting the complex is induced in response to oxidative stress [36]. To test this hypothesis and explore the functional role of PHB1 and PHB2 during ROS-mediated apoptosis, Kit225 cells were treated with 500 µM H_2_O_2_ for 0, 1, 3, 6, 12 and 24 hr. Hydrogen peroxide (H_2_O_2_) has been widely used as a ROS in various cell models as an inducer of apoptosis [44]. H_2_O_2_ is endogenously generated in the mitochondria and cytosol, and is one of the major contributors to oxidative damage [39].

**Figure 3:**
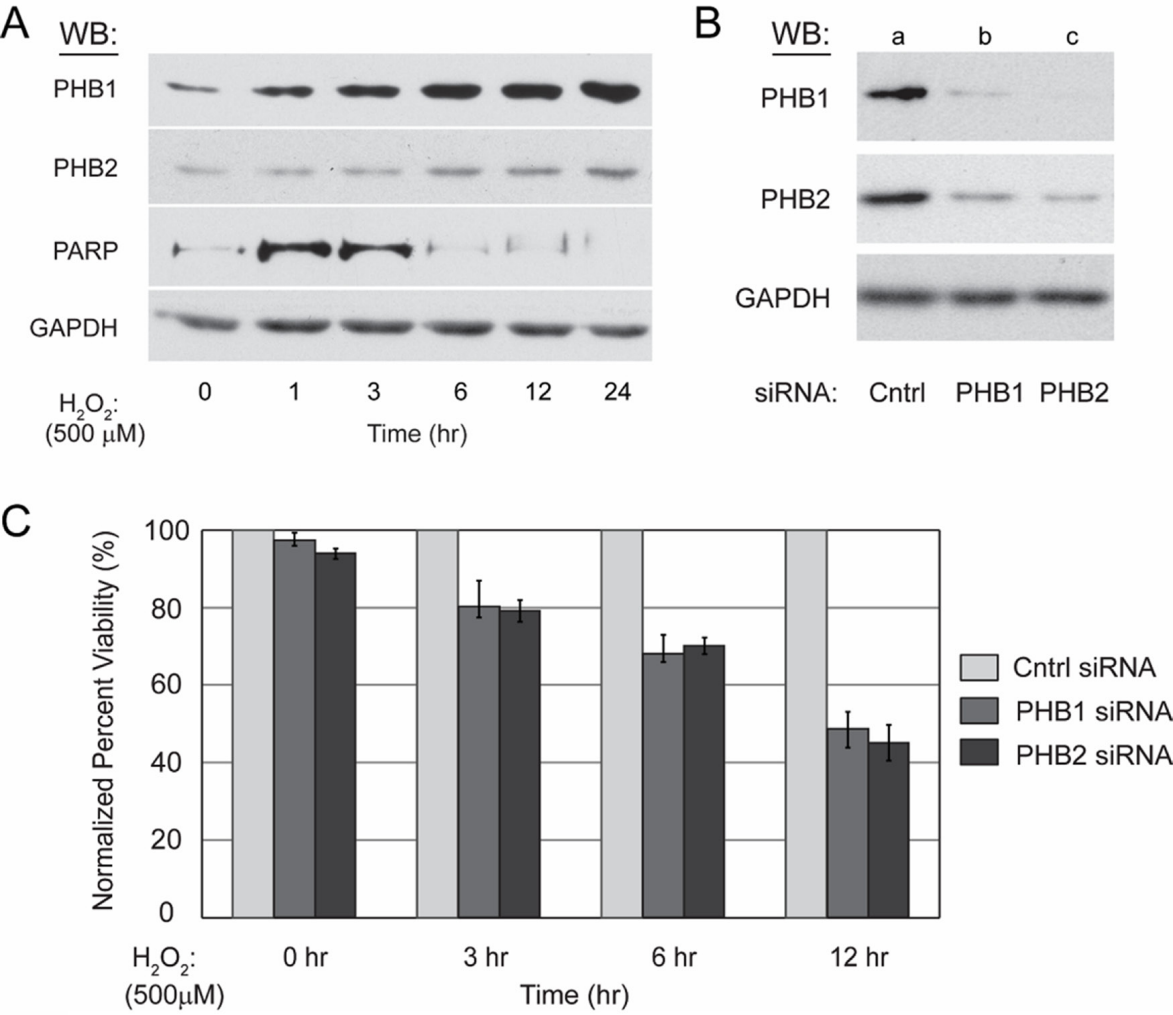
Kit225 cells unable to upregulate PHB1 and PHB2 are more sensitive to ROS-mediated cell death (**A**) Kit225 cells (5 × 10^6^) were treated with 500 µM H_2_O_2_ for 0, 1, 3, 6, 12 or 24 hr. Cell lysate (10 µg) was separated by 10% SDS-PAGE, transferred to PVDF membrane, and PHB1, PHB2, GAPDH and PARP protein levels detected by Western blot analysis as indicated. (**B**) Kit225 cells (5 × 10^6^) were electroporated with either non-targeting control siRNA (100 nM) (lane a), PHB1 specific siRNA (100 nM) (lane b), or PHB2 specific siRNA (100 nM) (lane c) and harvested at 48 hr post-transfection. Cell lysates (10 µg) were subjected to 10% SDS-PAGE and Western blot analysis with antibodies directed toward PHB1, PHB2 and GAPDH as indicated. (**C**) Kit225 (5 × 10^6^) cells were electroporated with control non-targeting siRNA (100 nM), PHB1 specific siRNA (100 nM), or PHB2 specific siRNA (100 nM), and treated with H_2_O_2_ (500 µM) for the indicated time points after 48 hr post-transfection with siRNA. Cell viability was determined by MTS assay. Values are the mean ± standard deviation of PHB1 and PHB2 siRNA treated percent viability normalized to control siRNA treated percent viability from three independent experiments.

Western blot analysis of protein lysate from treated Kit225 cells revealed PHB1 and PHB2 protein levels increase in a time dependent manner (Figure 3A). Detection of GAPDH protein levels was performed to confirm equal loading, whereas oxidative stress mediated caspase activation readily was verified by detection of caspase-cleaved PARP which reached maximum levels at approximately three hours.

The differential regulation of PHB1 and PHB2 during ROS-mediated cell death suggested the complex plays a functional role in the oxidative stress response of Kit225 cells. To determine the cellular consequences of PHB1 and PHB2 loss during ROS-mediated cell death, siRNA mediated knockdown was performed and cell viability monitored following a H_2_O_2_ treatment time course for 24 hrs. Kit225 cells were electroporated with non-targeting control siRNA (lane a), PHB1 specific siRNA (lane b), or PHB2 specific siRNA (lane c) and cultured for 48 hr before cell lysis, SDS-PAGE and Western blot detection of PHB1 and PHB2 to confirm knockdown (Figure 3B). The membrane was stripped and reprobed for GAPDH to confirm equal loading. An H_2_O_2_ time course was then performed after treatment with siRNA for 12 hr and cell viability determined by MTS assay at 0, 3, 6 and 12 hr after addition of H_2_O_2_. The percent viability of Kit225 cells following PHB1 and PHB2 depletion was 78%, 58% and 45% after 3, 6 and 12 hr H_2_O_2_ treatment, respectively, compared to control siRNA treated cells (Figure 3C). In summary, H_2_O_2_ treatment of Kit225 cells with diminished PHB1 and PHB2 expression displayed greater levels of cell death.

**Figure 4:**
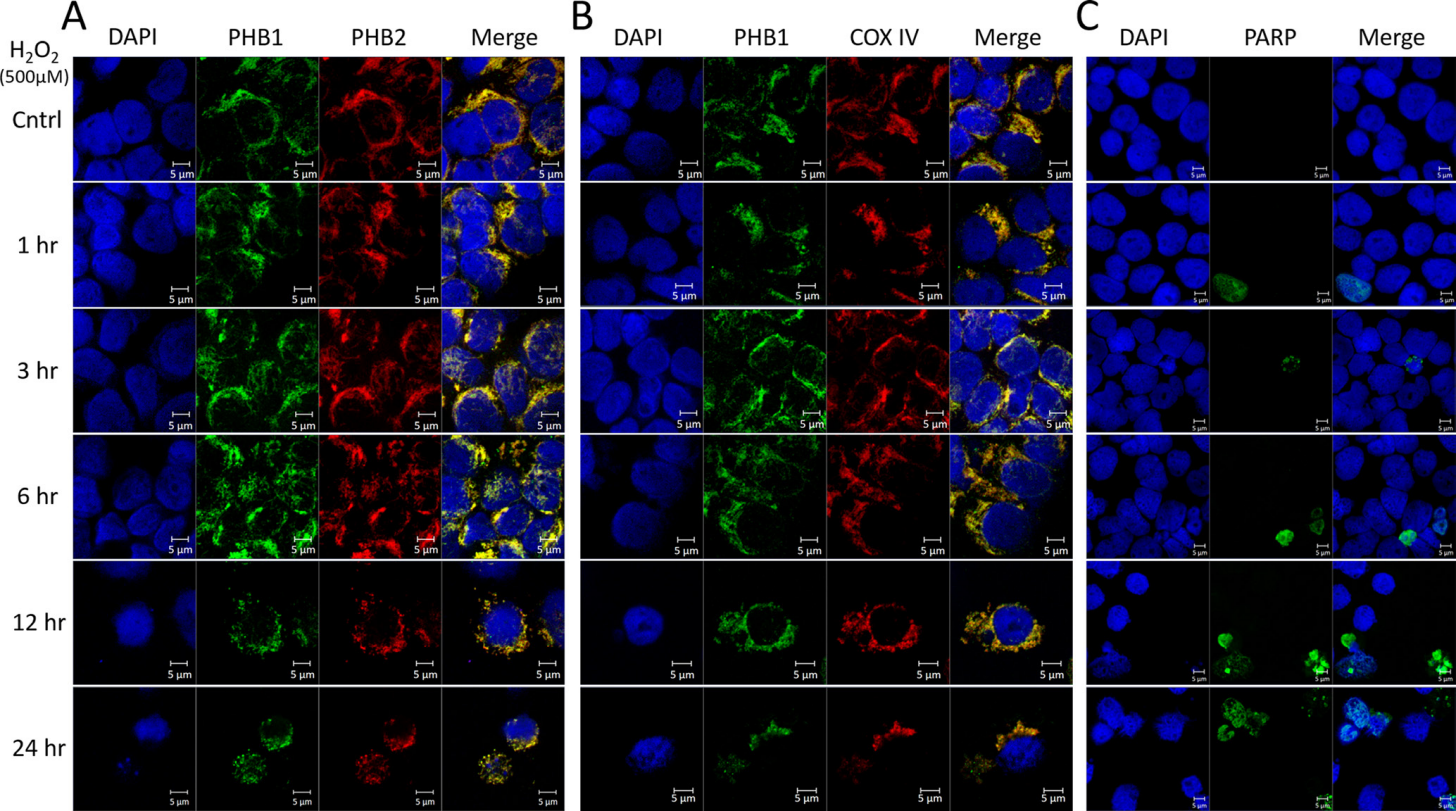
PHB1 and PHB2 co-localize to mitochondria of Kit225 during oxidative stress Kit225 cells were treated with 500 µM H_2_O_2_ for 0, 1, 3, 6, 12 or 24 hr. Cells were cytocentrifuged onto glass slides and subjected to analysis by immunofluorescent confocal microscopy. (**A**) PHB subcellular localization was determined by staining the nucleus with DAPI; PHB1-Alexa488 and PHB2-Cy3 and overlay (**B**) Nuclear staining with DAPI, PHB1-Alexa488, COX IV-Cy3 and overlay. (**C**) Nuclear staining with DAPI, cleaved PARP-Alexa488 and overlay were detected in Kit225 cells using immunofluorescent confocal microscopy.

To explore the subcellular localization of PHB1 and PHB2 during ROS-mediated apoptosis, Kit225 cells were treated with 500 µM H_2_O_2_ for 0, 1, 3, 6, 12 or 24 hr. Immunofluorescent confocal microscopy was utilized to evaluate PHB1 or PHB2 co-localization and prohibitin subcellular localization. In all treatments, PHB1 and PHB2 showed co-localization (Figure 4A). Similarly, PHB1 and the mitochondrial marker COX IV displayed co-localization at all time points tested (Figure 4B). Oxidative stress mediated caspase activation was verified via caspase-cleaved PARP (Figure 4C). Overall, the results suggest prohibitins are localized in the mitochondria during ROS-mediated cell death.

### PHB1 and PHB2 are overexpressed in tumor cells from patients diagnosed with lymphoid and myeloid malignancies

**Figure 5:**
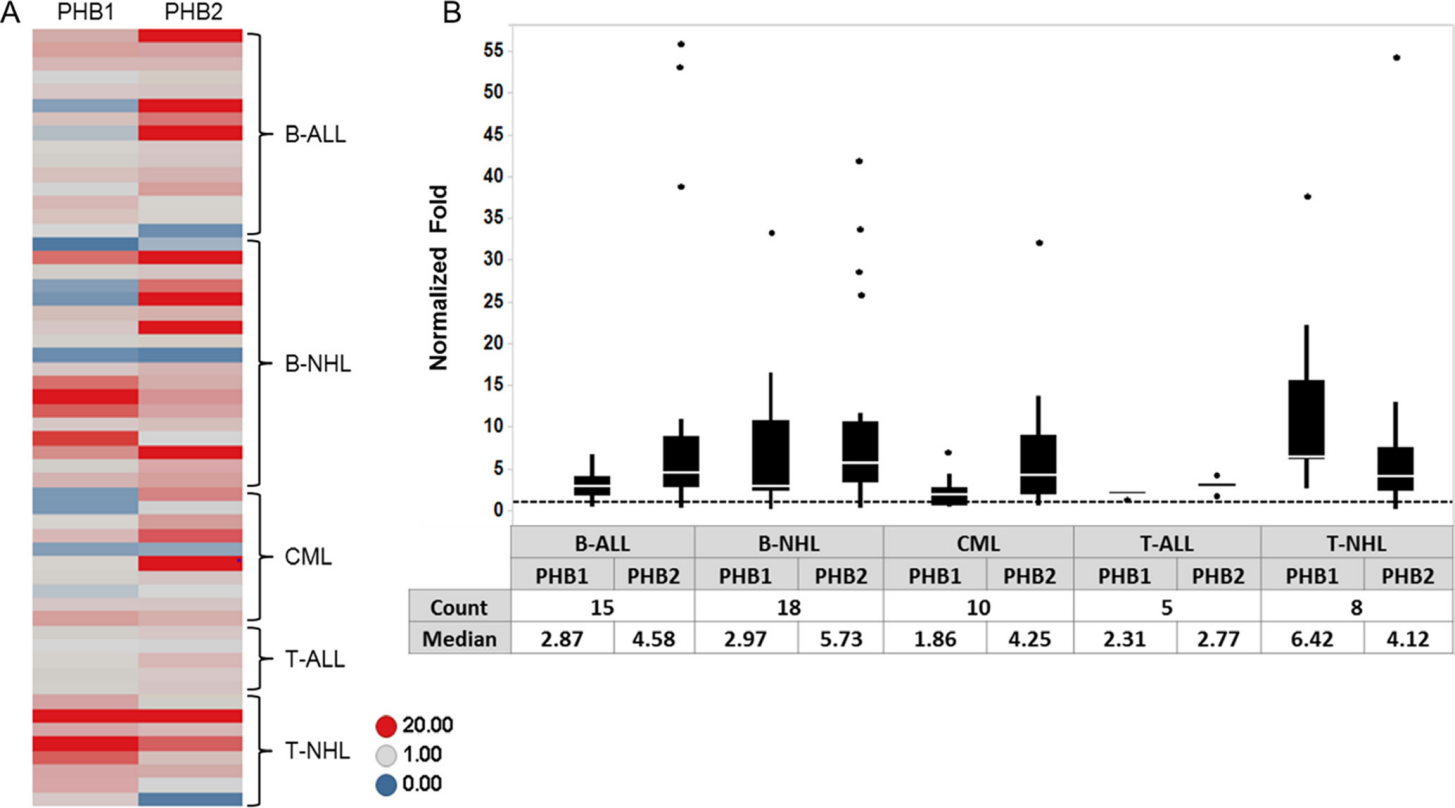
PHB1/PHB2 are overexpressed in tumor cells obtained from individuals diagnosed with lymphoid or myeloid malignancies PBMCs were isolated from normal donors (*n =* 5) and patients diagnosed with B-ALL (*n =* 15), B-NHL (*n =* 18), CML (*n =* 10), T-ALL (*n =* 5) and T-NHL (*n =* 8). Cell lysates (10 µg) were separated by 10% SDS-PAGE and subjected to Western blot analysis with antibodies directed to PHB1, PHB2 and GAPDH. (**A**) PHB1 and PHB2 band intensities were normalized to GAPDH using densitometric analysis and fold increase relative to the average expression in the normal PBMC donors plotted for each sample as a heat map. (**B**) Box plot depicting the relative fold expression of PHB1 and PHB2 across the different tumor cell types with the median fold increase denoted in the table (dots represent outlier values).

To further evaluate the expression of PHB1 and PHB2 in hematologic malignancies, Western blot analyses were performed on total cell lysates from PBMCs purified from healthy donors (*n =* 5) and tumor cells obtained from individuals diagnosed with B-ALL (*n =* 15), B-NHL (*n =* 18), CML (*n =* 10), T-ALL (*n =* 5) and T-NHL (*n =* 8). The membranes were stripped and reprobed for GAPDH levels to confirm equal loading. Densitometric analysis indicated that PHB1 and PHB2 were overexpressed (> 1 fold) in 61% and 79% of tumor samples, respectively compared to normal donor PBMCs (Figure 5A). The range of normalized prohibitin expression relative to normal PBMCs was 0.1 to 37.6 fold for PHB1 and 0.2 to 55.9 fold for PHB2 (Figure 5B). The median fold increase in PHB1/PHB2 protein expression in the B-ALL, B-NHL, CML, T-ALL, T-NHL patient samples were 2.9/4.6, 3/5.7, 1.9/4.3, 2.3/2.8, and 6.4/4.1 respectively (Figure 5B). Thus, concordant with prohibitin expression observed in tumor cell lines (Figure 1), PHB1 and PHB2 are overexpressed in lymphoid (T- and B-cell leukemia/lymphoma) and myeloid (CML) primary patient tumor cells.

### PHB1 and PHB2 co-localize to the mitochondria in primary hematologic tumor cells

**Figure 6:**
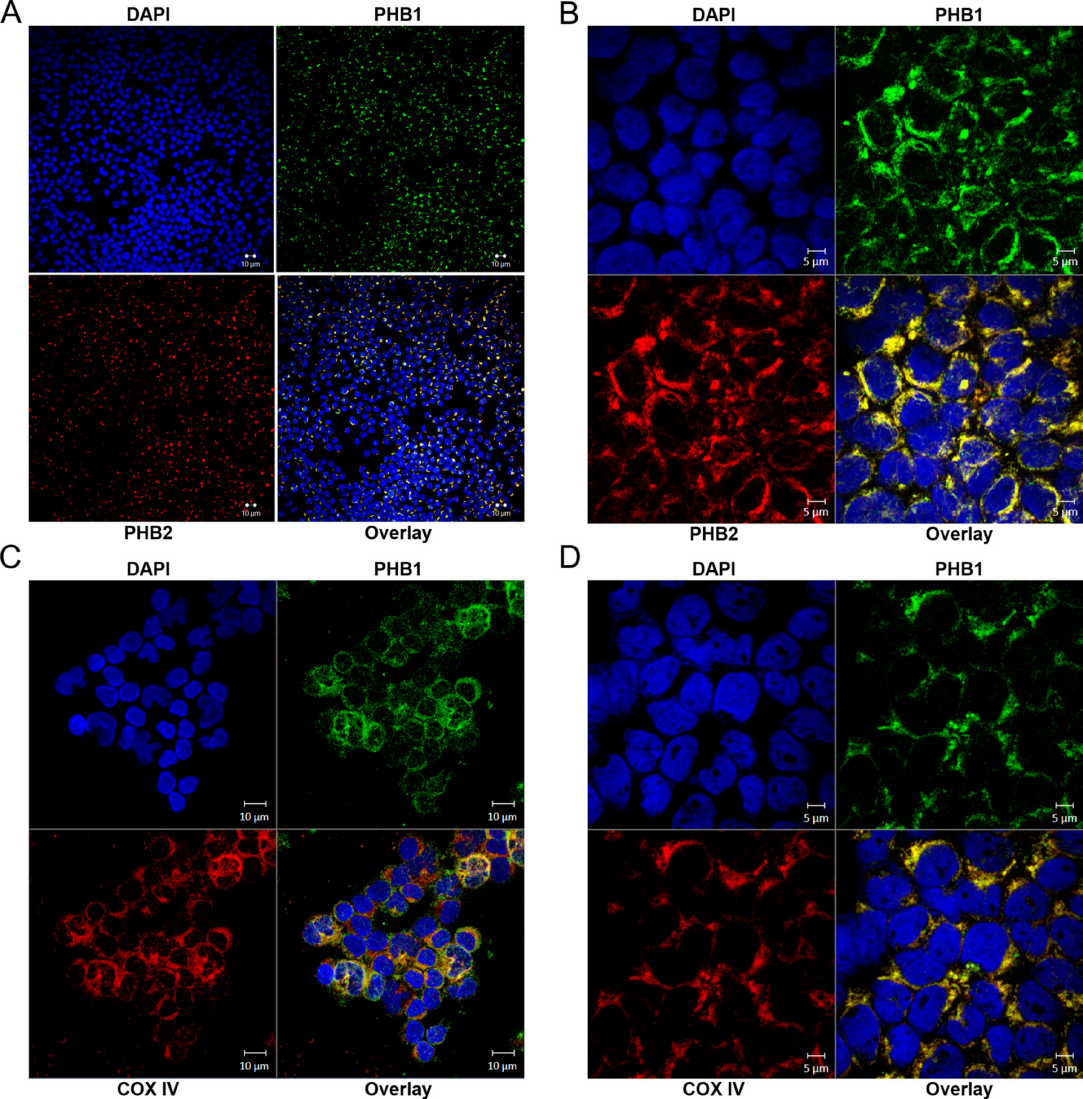
PHB1/PHB2 co-localize to peri-nuclear regions and mitochondria in tumor cells obtained from patients diagnosed with hematologic malignancies Samples were cytocentrifuged onto glass slides and subjected to analysis by immunofluorescent confocal microscopy. Immunofluorescent confocal microscopy displaying DAPI (upper left panels); PHB1-Cy2 (upper right panels), PHB2-Cy3 or COX IV-Cy3 (lower left panels) and overlay (lower right panels) in (**A** and **C**) T-ALL and (**B** and **D**) CML patient samples.

To determine PHB1 and PHB2 localization in primary hematologic tumor cells, dual labeled immunofluorescent confocal microscopy was utilized (Figure 6). Similar to prohibitin localization in normal PBMCs [36] and Kit225 cells (Figure 2), PHB1 and PHB2 co-localize primarily to peri-nuclear regions with a specific punctuate staining pattern, indicative of mitochondrial localization, in T-ALL (Figure 6A) and CML (Figure 6B). There were no detectable levels of PHB1 or PHB2 observed at the plasma membrane or in the nucleus. Likewise, subcellular localization was evaluated using the inner mitochondrial membrane marker COX IV (Figure 6D). Immunofluorescent confocal microscopy detecting PHB1 and COX IV showed co-localization of PHB1 with COX IV in T-ALL and CML. These findings further support the role of the prohibitin complex in promoting hematologic tumor cell survival through mitochondrial stabilization.

## DISCUSSION

In an effort to gain insight into the prohibitin molecular mechanism of action in lymphoid and myeloid-derived malignancies, PHB1 and PHB2 were determined to be overexpressed in tumor cell lines compared to normal primary human PBMCs (Figure 1) and localized primarily to the mitochondria in Kit225 cells (Figure 2). siRNA mediated knockdown of PHB1 and PHB2 in Kit225 cells significantly enhanced their sensitivity to ROS-induced cell death, suggesting a protective function in human hematologic tumor cells (Figure 3). This finding was substantiated by evidence demonstrating PHB1 and PHB2 are upregulated during ROS-mediated apoptosis (Figure 3). Moreover, PHB1 and PHB2 protein levels were significantly higher in tumor cells isolated from leukemia and lymphoma patients compared to healthy donor PBMCs (Figure 5) and localized to primarily to the mitochondria (Figure 6). Taken together, these findings support the role of PHB1 and PHB2 in hematologic tumor cells for maintenance of mitochondrial integrity, which may facilitate the energy requirements of these tumor cells. Therefore, prohibitins serve as novel biomarkers and putative molecular targets for therapeutic intervention in lymphoid and myeloid malignancies.

Prohibitins have been proposed to play key roles in a variety of disease states, however their function in lymphocytes has not been clearly defined [24]. Evidence is provided herein that PHB1 and PHB2 are overexpressed in a panel of leukemia and lymphoma cell lines compared to normal naïve PBMCs (Figure 1). Likewise, primary tumor cells obtained from leukemia and lymphoma patients displayed similar levels of overexpressed prohibitins (Figure 5). Although prohibitins have been reported to localize to a variety of subcellular locations in tumor cells [45, 46], PHB1 and PHB2 were shown to co-localize primarily to the mitochondria in Kit225 and leukemia/lymphoma patient tumor cells (Figures 2 and 6). Furthermore, PHB1 and PHB2 were upregulated and co-localized in mitochondria upon oxidative stress induced cell death. This data suggests both proteins play a protective or anti-apoptotic function in lymphoid tumor cells (Figures 3 and 4). PHB1 and PHB2 knockdown significantly enhanced the susceptibility of Kit225 cells to ROS-induced cell death (Figure 3). The mitochondrial chaperone action of PHB1 and PHB2 supports the hypothesis that prohibitins can play a protective function for cells undergoing transformation.

Mitochondrial dysfunction is a hallmark of cellular transformation and increasing evidence supports its importance in human pathology. It is well established that cancer cells have an enhanced energy demands including an increase in glycolysis, glucose transport, gluconeogenesis and pentose phosphate pathway activity [47–49]. Considering the critical role of chaperones in the maintenance of mitochondrial integrity, it is reasonable to expect prohibitin overexpression is functionally linked to oncogenesis. Indeed, PHB1 and PHB2 protein levels were overexpressed in tumor cells isolated from patients with leukemia and lymphoma compared to normal naïve PBMCs (Figure 5). Previous reports suggest PHB1 mRNA levels are inversely proportional to cellular proliferation in a number of cell types [20, 50, 51], however the results presented herein indicate PHB1 and PHB2 are upregulated during the oxidative stress response in hematologic tumor cells.

The regulation of prohibitins in response to various stimuli has been reported in a number of tumor cell types. Luan et al. established that PHB1 knockdown reverses the “epithelial-to-mesenchymal transition” phenotype in pancreatic cancer cell lines. It was also concluded that disrupting the Ras/Raf/MEK/ERK pathway by blocking Prohibitin-cRaf interaction diminished the viability of pancreatic cancer cells *in vitro,* and inhibits their migration *in vitro* and *in vivo* [26]. Moreover, phosphorylation of PHB1 at T258 on the plasma membrane activates PI3K/AKT and the Ras/Raf/MEK/ERK pathways promoting proliferation and metastasis of cancer cells in lung and cervical adenocarcinoma cell lines [52]. Additionally, the oncoprotein c-Myc, whose activation and deregulation by chromosomal translocations is a major feature of certain leukemias and lymphomas [53], was shown to target the PHB1 gene by binding and inducing its transcription on a specific consensus sequence [54]. This interplay of oncogenic signaling molecules suggests that one mechanism of cellular transformation occurs through the upregulation of prohibitins to protect mitochondria during the increased bioenergetic requirements demanded by tumor cells. Similarly, interleukin 6 signaling through STAT3 was shown to modulate PHB1 expression in intestinal epithelial cells where it was shown to protect against oxidative stress [55]. The pathophysiological role of the JAK/STAT signaling pathway in hematologic malignancies is well established [54, 56, 57]. In conclusion, these observations indicate that PHB1 and PHB2 activity contribute to tumor cell survival in the context of mitochondrial protection and therefore strengthen the potential value of these proteins as therapeutic targets in the treatment hematologic cancers.

## MATERIALS AND METHODS

### Cell culture and patient samples

Kit225 cells were maintained in RPMI 1640 supplemented with 10% FCS (Atlanta Biologicals), 2 mM L-glutamine, 50 IU/ml penicillin, and 50 mg/ml streptomycin (complete RPMI) plus 10 IU/ml recombinant IL-2. Jurkat, MT-2, Hut102, HH, H9, YT, KCL-22, Raji, Ramos, BJAB, Nalm-6 and CCRF-CEM cells were maintained in complete RPMI without IL-2.

Human peripheral blood mononuclear cells (PBMCs) were obtained from healthy donors, purified by isocentrifugation, and activated with phytohemagglutinin (PHA) (1 μg/ml) for 72 hr, as previously described [36]. Primary patient leukemia and lymphoma cells were obtained from de-identified excess diagnostic material (peripheral blood, bone marrow or lymph node biopsies) through a The University of Texas at El Paso Institutional Review Board approved study. All clinical materials were obtained with the patients’ consent and approval from the local ethics committee.

### Cell lysis and western blot analysis

Cell pellets were solubilized in 1% Triton X-100 containing lysis buffer and Western blot analysis was performed as previously described using the following antibodies [58]: affinity purified rabbit polyclonal anti-PHB2 [36], monoclonal anti-PHB1 (Abcam), monoclonal anti-actin (Sigma Aldrich), and monoclonal anti-GAPDH (Fitzgerald). For all samples, total protein was determined by the bicinchoninic acid method (Pierce Biotech).

### Reactive oxygen species (ROS)-induced apoptosis

For induction of ROS-mediated apoptosis, Kit225 cells were treated with hydrogen peroxide (H_2_O_2_) (500 µM) (Sigma Aldrich) at 37°C at the time points indicated and cellular viability evaluated. Additionally, caspase activation was determined by detection of PARP cleavage by Western blot analysis with rabbit polyclonal anti-PARP (Cell Signaling), which recognizes full length (116 kDa) as well as the large (89 kDa) cleavage fragment.

### Viability assay

Cell viability was assessed by using the MTS (3-(4,5-dimethylthiazol-2-yl)-5-(3-carboxymethoxyphenyl)-2-(4-sulfophenyl)-2H-tetrazolium) reagent (Promega), following the manufacturer’s instructions. Cells were seeded in flat-bottom tissue culture 96-well plates at a density of 100,000 per well in a 100 µl of culture media. After the indicated times of incubation, 20 µl of the MTS reagent were added to each well containing the cells, followed by an additional incubation for 30 min. To stop the reaction we add 25 ul 10% SDS. The purple water-soluble formazan product was measured by absorbance at 490 nm utilizing a microplate reader (VERSAmax tunable microplate reader), as previously described [59]. Two independent experiments, each performed in triplicate, were performed. Data are shown as a normalized percentage of cell viability and are consistently reported as the average with the corresponding standard deviations.

### Immunofluorescent confocal microscopy

Kit225 and primary patient tumor cells were cytocentrifuged onto glass slides, fixed with cold methanol and permeabilized with 0.2% Triton X-100. The cell staining procedures were performed using the following antibodies as previously described [36]: mouse monoclonal anti-PHB1 (abcam), affinity purified rabbit polyclonal anti-PHB2 [36], mouse monoclonal anti-OxPhos CII (Invitrogen), or rabbit monoclonal anti-COX IV (cell signaling). To reduce photobleaching effects, all staining steps were executed in the dark. High-resolution digital fluorescent images were captured from the stained cells using an LSM 700 confocal laser scanning microscope equipped with a 40× and 63× immersion oil objective (Zeiss, New York, NY). Image acquisition was performed in the multitrack scanning mode with the excitation wavelength at 405, 488 and 555 nm, corresponding to the blue, green and red fluorescence signals, respectively. Consistently, the confocal images were acquired with the same settings and analyzed using the ZEN 2009 software (Zeiss). Collected images were exported in a 12-bit TIFF RGB format.

### siRNA mediated silencing of PHB1 and PHB2

PHB1 (SMARTpool Cat. # M-010530-00), PHB2 (SMARTpool Cat. # M-018703-00) and control non-targeting (siControl pool Cat. # D-001206-13) siRNA were purchased from Dharmacon. Transfection of Kit225 cells was carried out by electroporation using the Nucleofection system (Amaxa). Briefly, Kit225 cells (5 × 10^6^) were suspended in 100 μl of transfection solution V and transfected with 1.5 μg of PHB1, PHB2 or control siRNA using the X-001 program. Transfected cells were immediately diluted with pre-warmed complete RPMI containing interleukin 2 (10 IU/ml) and cultured for the time indicated.
